# Supplementary material for: End Sequence Analysis Toolkit (ESAT) expands the extractable information from single-cell RNA-seq data
Source: Genome Res. 2016 Oct;26(10):1397–410. doi: 10.1101/gr.207902.116 (PMC5052061; doi:10.1101/gr.207902.116)
Supplement: Supplemental Material [file supp_gr.207902.116_Supp_Materials.tar.gz › ESAT-esat_v0.1/doc/html/classbroad_1_1pda_1_1seq_1_1clip_1_1_fasta_extend_peaks.html]

ESAT: broad.pda.seq.clip.FastaExtendPeaks Class Reference


ESAT


- Main Page
- Related Pages
- Packages
- Classes
- Files

- Class List
- Class Index
- Class Hierarchy
- Class Members

All Classes Namespaces Files Functions Variables Enumerator Pages

- broad
- pda
- seq
- clip
- FastaExtendPeaks

Public Member Functions |
Static Public Member Functions |
List of all members

broad.pda.seq.clip.FastaExtendPeaks Class Reference

|  |  |
| --- | --- |
| Public Member Functions | |
|  | FastaExtendPeaks (String genomeFasta, String bedFile, String peakFile, int windowSize) throws IOException |
|  | |
|  | FastaExtendPeaks (Map< String, Sequence > chrsByName, String bedFile, String peakFile, int windowSize) throws IOException |
|  | |
|  | FastaExtendPeaks (Map< String, Sequence > chrsByName, String bedFile) throws IOException |
|  | |
|  | FastaExtendPeaks (Map< String, Sequence > chrsByName, String bedFile, String peakFile) throws IOException |
|  | |
|  | FastaExtendPeaks (String genomeFasta, String bedFile) throws IOException |
|  | |
| void | writeFasta (Map< String, Collection< Gene >> genesToWrite, String outFile) throws IOException |
|  | |

|  |  |
| --- | --- |
| Static Public Member Functions | |
| static void | main (String[] args) throws IOException |
|  | |

## Detailed Description

Author
:   shari Extends peaks to standardized size (for probe design, etc.)

## Constructor & Destructor Documentation

|  |  |  |  |
| --- | --- | --- | --- |
| broad.pda.seq.clip.FastaExtendPeaks.FastaExtendPeaks | ( | String | *genomeFasta*, |
|  |  | String | *bedFile*, |
|  |  | String | *peakFile*, |
|  |  | int | *windowSize* |
|  | ) |  | throws IOException |

Parameters
:   |  |  |
    | --- | --- |
    | genomeFasta | Fasta file of sequences |
    | bedFile | Bed file |

Exceptions
:   |  |  |
    | --- | --- |
    | IOException |  |

Here is the caller graph for this function:

|  |  |  |  |
| --- | --- | --- | --- |
| broad.pda.seq.clip.FastaExtendPeaks.FastaExtendPeaks | ( | Map< String, Sequence > | *chrsByName*, |
|  |  | String | *bedFile*, |
|  |  | String | *peakFile*, |
|  |  | int | *windowSize* |
|  | ) |  | throws IOException |

Load sequences and annotations

Parameters
:   |  |  |
    | --- | --- |
    | chrsByName | Chromosomes by name |
    | bedFile | Genes in bed format |
    | windowSize | size of return sequences |

Exceptions
:   |  |  |
    | --- | --- |
    | IOException |  |

|  |  |  |  |
| --- | --- | --- | --- |
| broad.pda.seq.clip.FastaExtendPeaks.FastaExtendPeaks | ( | Map< String, Sequence > | *chrsByName*, |
|  |  | String | *bedFile* |
|  | ) |  | throws IOException |

Load sequences and annotations

Parameters
:   |  |  |
    | --- | --- |
    | chrsByName | Chromosomes by name |
    | bedFile | Genes in bed format |

Exceptions
:   |  |  |
    | --- | --- |
    | IOException |  |

|  |  |  |  |
| --- | --- | --- | --- |
| broad.pda.seq.clip.FastaExtendPeaks.FastaExtendPeaks | ( | Map< String, Sequence > | *chrsByName*, |
|  |  | String | *bedFile*, |
|  |  | String | *peakFile* |
|  | ) |  | throws IOException |

|  |  |  |  |
| --- | --- | --- | --- |
| broad.pda.seq.clip.FastaExtendPeaks.FastaExtendPeaks | ( | String | *genomeFasta*, |
|  |  | String | *bedFile* |
|  | ) |  | throws IOException |

## Member Function Documentation

|  |  |  |  |  |  |
| --- | --- | --- | --- | --- | --- |
| static void broad.pda.seq.clip.FastaExtendPeaks.main | ( | String[] | *args* | ) | throws IOException |

static

Parameters
:   |  |  |
    | --- | --- |
    | args |  |

Exceptions
:   |  |  |
    | --- | --- |
    | IOException |  |

Here is the call graph for this function:

|  |  |  |  |
| --- | --- | --- | --- |
| void broad.pda.seq.clip.FastaExtendPeaks.writeFasta | ( | Map< String, Collection< Gene >> | *genesToWrite*, |
|  |  | String | *outFile* |
|  | ) |  | throws IOException |

Parameters
:   |  |  |
    | --- | --- |
    | outFile |  |

Exceptions
:   |  |  |
    | --- | --- |
    | IOException |  |

---

The documentation for this class was generated from the following file:

- java/broad/pda/seq/clip/FastaExtendPeaks.java


---

Generated on Fri Aug 15 2014 15:11:01 for ESAT by  

 1.8.7
